# Supplementary material for: Role of Advanced Glycation End-Products and Other Ligands for AGE Receptors in Thyroid Cancer Progression
Source: J Clin Med. 2021 Sep 10;10(18):4084. doi: 10.3390/jcm10184084 (PMC8470575; doi:10.3390/jcm10184084)
Supplement: Supplementary file 1 [file jcm-10-04084-s001.zip › jcm-1322052-SI.pdf]

Table S1. The etiology, characteristics, progression, diagnosis and treatment of thyroid cancers: PTC, FTC, ATC, PDTC, MTC, PTL, PTS and STCT.

Type of thyroid cancer	Prevalence among thyroid carcinomas	Oncogens	Chromosomal rearrangements	Carcinoma characteristics	Carcinoma progression	Diagnosis	Treatment	References
Papillary thyroid cancer (PTC)	70% – 90% of TC, often in young women and girls at adolescence	$BRAF^{V600E}$<br>NRAS<br>HRAS<br>KRAS<br>EIF1AX<br>PIK3CA<br>PTEN/PHTS<br>$SPOP^{SCR3}$<br>TP53<br>$TERT^{C228T}$	RET with: ACBD5, AFAP1L2, AKAP13, ANKRD2, PTC1, DLG5, ERC1, FKBP15, PTC5, HOOK3, KIAA1468, PTC8, PTC3, PCM1, PTC2, RUFY2, SPECC1L, TBL1XR1, SQSTM1, PTC6, TRIM27, PTC7 or UEVLD<br><br>PAX8 with PPARG	tumor: irregular borderline, surrounded by capsule, tendency to express the biggest undiversity of stroma among TC, prognosticates well.<br>Aggressive variants: columnar tumour, tall cell tumour, solid tumour and hobnail .	slow growth, metastases within thyroid flesh and to lymph nodes, remains iodine catching.	problematic, similar to benign tubercle of thyroid gland with papillary hyperplasia (BTN-PH)	removal of the thyroid gland along with regional lymph nodes, in the case of papillary thyroid microcarcinoma - regular USG and TSH monitoring	[1–7]
Folicullar thyroid	10% – 15% of TC,	$BRAF^{K601E}$<br>NRAS<br>HRAS	PAX8 with PPARG	microfolicullar growth or solid/ trabecular: in ~	slow growth, metastases to bones and	similar to an adenoma, commonly	Total resection of the one lobe with isthmus	[8–15]

carcinoma (FTC)	more aggressive than PTC, often in women (40 – 60 years old)	KRAS<br>EIF1AX<br>PIK3CA<br>PTEN/PHTS<br>DICER1<br>$EZH1^{Q571R}$<br>$EZH1^{Y642F}$<br>$EZH1^{M349L}$<br>TP53<br>$TERT^{C250T}$		80% of FTC cases; follicles containing colloid: in 20% of FTC cases, usually well formed capsule with blood vessels	lungs, it is iodine-catching, more expansive invasion of capsule and extension of FTC into thyroid flesh - higher metastases rate and death rate	diagnosed without precise assessment of lesion's malignancy, diagnosis stated on the basis of USG with aimed biopsy is difficult. Sign of malignancy: the invasion to vessels and tumour capsule.	with removal of regional lymph nodes, opinions concerning. Expanse of operation are disparat. Lowering of operation expanse - lower risk of complications. Exception acceptable to refrain from operation : autonomous tumours in scyntygraphy, small follicular tubercles, controlled by biopsies.	
Anaplastic thyroid carcinoma (ATC)	less than 2% of TC, 50% of mortality in TC, most frequently in the elderly	TP53<br>$BRAF^{V600E}$<br>NRAS<br>HRAS<br>KRAS<br>EIF1AX<br>PIK3CA<br>$TERT^{C250T}$	PAX8 with PPARG	the most heterogenous of TC, a broad spectrum of differentiation – tumours built by fusiform cells, pleomorphic giant cells and epithelial cells, ATC stroma:	aggressive, malignant tumour, fast metastases to distant organs, early metastases via bloodstream and lymphatic	diagnosis is difficult - morphological overlapping with other cancers of anaplastic morphology, similar to: fibrosarcoma, undifferentiated	conventional treatment methods are not successful, radical surgery is often impossible, chemotheraphy : doxorubicin monotherapy	[16–20]

				changingly hyaloid, hardened or desmoplastic, well defined oncological capsule is an important prognostic factor in ATC - lengthen survival time,	system, it is not iodine-catching, prognosticates badly	pleomorphic sarcoma, single fibrous tumour, angiosarcoma or rhabdomyosarcoma, potential diagnosis marker: TP53 mutation, it occurs in about 70% of ATC cases, feature specific for ATC	or multi-drug regimens	
Poorly differentiated thyroid carcinoma (PDTC)	2% – 5% of TC, in people over 60 years of age, more often in men, a poor prognosis, relapse-free time is shorter than 12 months	$BRAF^{V600E}$<br>NRAS<br>HRAS<br>KRAS<br>EIF1AX<br>TP53<br>$TERT^{C250T}$	RET with: TFG, PTC1, PTC3 or PPFIBP2	monomorphic cancer cells form heterogeneous structure of islet, trabecular and solid build, sometimes satellite nodes, rare colloidal tumour surrounded tightly by solid tumour with necrosis	Expansive growth and partial capsule, metastasis into regional lymph nodes and into distant organs	the majority of cell tumours exhibit poorly differentiated features, PDTC displays predominant solid, insular, or trabecular growth patterns sometimes coexist with follicular, papillary or anaplastic carcinoma component<br><br>smaller lesions of lymphocytes and dendritic cells, higher number of macrophages	surgical removal of the thyroid gland and treatment with reactive iodine	[21–25]

Medullary thyroid carcinoma (MTC)	5% – 10% of TC, more frequent in women, may occur in children (below than 10% of cases), prognosticates unsurely	NRAS HRAS KRAS	RET with MYH13	heterogenous cancer with differentiation of parafollicular and follicular cell lines; deriving from C cells (main source of calcitonin in the body; excrete also: carcinoembrionic antygen (CEA), amyloid, adrenocorticotropic hormone (ACTH), katacalcin, calcitonin-gene related peptide (CGRP), somatostatin, vasoactive intestinal peptide (VIP), prostaglandins, histaminase, chromogranin A, serotonin, neurospecific enolase and melanin), occur sporadicly or can accompany congenital	tumor is hard, possesses no capsule, it is white or red, located mainly in 2/3 of back-upper part of thyroid gland, advanced cancerous lesion affect trachea, recurrent laryngeal nerve and surrounding tissues, distant metastases into: lymph nodes, liver, lungs and bones, is not iodine-catching,	round, polymorphic or fusiform cells forming nests separated from one another by connective tissue are visible in histopatological image	excision of the thyroid gland and central lymph nodes of the neck, and in the case of a tumor> 1 cm in diameter, the lateral lymphatic system of the neck is usually removed from the side of the tumor, in inoperable tumors - Isotope therapy with iodine 131, chemotherapy are complementary treatments	[26–31]

				multiple endocrine neoplasia type 2 (20- 30% of cases)				
Primary thyroid lymphoma (PTL)	Less than 5% of TC, more common in females (over 65 years of age)		IGH with BCL2	Causes by proliferation of B lymphocytes	suspicion of thyroid lymphoma - rapidly growing mass in the neck accompanyin g Hashimoto inflammation,	rare disease, difficult to diagnose, tumor: huge, unilateral mass, hipoechogenic, invading neighbouring soft tissues	treatment depends on its grade and location, low-grade lymphomas are treated with radiotherapy; high-grade lymphomas with chemotherapy. Monoclonal antibodies are also used. The surgical removal of the entire thyroid is rare.	[32–34]
Primary thyroid sarcoma (PTS)	Less than 1.5% of TC, often in young adults, 4- times more frequently in men		BCOR with CCNB3	clinical and pathological features are similar to sarcoma of other organs,	a painless goiter, infiltration of adjacent organs (trachea or esophagus), metastatic invasion of local lymph	diagnostic rarity - difficulties in setting the correct diagnosis,	adjuvant chemotherapy before surgery or radiotherapy, a broad spectrum of treatment strategies: surgery, with or without additional	[35–37]

					nodes, distal metastases		chemotherapy and local radiation, palliative care.	
Squamous-cell thyroid carcinoma (SCTC)	Less than 1 % of TC, often in women (over 60 years of age), very aggressive tumor with a poor prognosis			differentiated, poorly understood	a primary or secondary disease, rich vascular nature but low the incidence of metastatic thyroid disease (2-3%),	radiological imaging from the neck to the abdomen (to exclude secondary diseases),	surgical resection of the tumor with adjuvant radiotherapy and chemotherapy is the recommended option – often resistant to radiotherapy,	[38,39]

1. Bouchard, J.J.; Otero, J.H.; Scott, D.C.; Szulc, E.; Martin, E.W.; Sabri, N.; Granata, D.; Marzahn, M.R.; Lindorff-Larsen, K.; Salvatella, X.; et al. Cancer Mutations of the Tumor Suppressor SPOP Disrupt the Formation of Active, Phase-Separated Compartments. *Mol. Cell* **2018**, *72*, 19-36.e8, doi:10.1016/j.molcel.2018.08.027.

2. Gonzalez-Gonzalez, R.; Bologna-Molina, R.; Carreon-Burciaga, R.G.; Gómezpalacio-Gastelum, M.; Molina-Frechero, N.; Salazar-Rodríguez, S. Papillary Thyroid Carcinoma: Differential Diagnosis and Prognostic Values of Its Different Variants: Review of the Literature. *ISRN Oncol.* **2011**, *2011*, 1–9, doi:10.5402/2011/915925.

3. Nath, M.C.; Erickson, L.A. Aggressive Variants of Papillary Thyroid Carcinoma: Hobnail, Tall Cell, Columnar, and Solid. *Adv. Anat. Pathol.* **2018**, *25*, 172–179, doi:10.1097/PAP.0000000000000184.

4. Qiu, Z.L.; Shen, C.T.; Sun, Z.K.; Wei, W.J.; Zhang, X.Y.; Song, H.J.; Luo, Q.Y. Circulating Long Non-Coding RNAs Act as Biomarkers for Predicting 131 I Uptake and Mortality in Papillary Thyroid Cancer Patients with Lung Metastases. *Cell. Physiol. Biochem.* **2016**, *40*, 1377–1390, doi:10.1159/000453190.

5. Levy, R.A.; Hui, V.W.; Sood, R.; Fish, S.; Markowitz, A.J.; Wong, R.J.; Guillem, J.G. Cribriform-morular variant of papillary thyroid

carcinoma: an indication to screen for occult FAP. *Fam. Cancer* **2014**, *13*, 547–551, doi:10.1007/s10689-014-9732-5.

6. Laforga, J.B.; Pedro, T.; Gasent, J.M. Pulmonary metastasis of cribriform-morular variant of thyroid carcinoma mimicking primary adenocarcinoma of the lung: A potential pitfall. *Diagn. Cytopathol.* **2020**, *48*, 78–81, doi:10.1002/dc.24312.

7. Santoro, M.; Moccia, M.; Federico, G.; Carlomagno, F. RET Gene Fusions in Malignancies of the Thyroid and Other Tissues., doi:10.3390/genes11040424.

8. Poller, D.N.; Megadmi, H.; Ward, M.J.A.; Trimboli, P. Hürthle cells on fine-needle aspiration cytology are important for risk assessment of focally pet/ct fdg avid thyroid nodules. *Cancers (Basel).* **2020**, *12*, 1–9, doi:10.3390/cancers12123544.

9. Jung, S.H.; Kim, M.S.; Jung, C.K.; Park, H.C.; Kim, S.Y.; Liu, J.; Bae, J.S.; Lee, S.H.; Kim, T.M.; Lee, S.H.; et al. Mutational burdens and evolutionary ages of thyroid follicular adenoma are comparable to those of follicular carcinoma. *Oncotarget* **2016**, *7*, 69638–69648, doi:10.18632/oncotarget.11922.

10. Baloch, Z.W.; LiVolsi, V.A. Follicular-patterned lesions of the thyroid: The bane of the pathologist. *Am. J. Clin. Pathol.* 2002, *117*, 143–150.

11. Gupta, N.; Dasyam, A.K.; Carty, S.E.; Nikiforova, M.N.; Ohori, N.P.; Armstrong, M.; Yip, L.; LeBeau, S.O.; McCoy, K.L.; Coyne, C.; et al. RAS mutations in thyroid FNA specimens are highly predictive of predominantly low-risk follicular-pattern cancers. *J. Clin. Endocrinol. Metab.* **2013**, *98*, doi:10.1210/jc.2012-3396.

12. Follicular carcinoma of the thyroid: an evaluation of the histologic criteria used for diagnosis - PubMed Available online: https://pubmed.ncbi.nlm.nih.gov/6877876/ (accessed on Mar 10, 2021).

13. Dratwa, M.; Wysoczańska, B.; Łacina, P.; Kubik, T.; Bogunia-Kubik, K. TERT—Regulation and Roles in Cancer Formation. *Front. Immunol.* 2020, *11*.

14. Ravella, L.; Lopez, • J; Descotes, • F; Giai, • J; Lapras, • V; Denier, M.-L.; Borson-Chazot, • F; Lifante, J.-C.; Decaussin-Petrucci, • M; Decaussin-Petrucci, M. Preoperative Role of RAS or BRAF K601E in the Guidance of Surgery for Indeterminate Thyroid Nodules. *World J. Surg.* **2020**, *44*, 2264–2271, doi:10.1007/s00268-020-05487-1.

15. Calebiro, D.; Grassi, E.S.; Eszlinger, M.; Ronchi, C.L.; Godbole, A.; Bathon, K.; Guizzardi, F.; De Filippis, T.; Krohn, K.; Jaeschke, H.; et al. Recurrent EZH1 mutations are a second hit in autonomous thyroid adenomas. *J. Clin. Invest.* **2016**, *126*, 3383–3388, doi:10.1172/JCI84894.

16. Thyroid gland undifferentiated (anaplastic) carcinoma (Concept Id: C0238461) - MedGen - NCBI Available online:

https://www.ncbi.nlm.nih.gov/medgen/116064 (accessed on Mar 10, 2021).

17. Yin, Y.; Hong, S.; Yu, S.; Huang, Y.; Chen, S.; Liu, Y.; Zhang, Q.; Li, Y.; Xiao, H. MiR-195 Inhibits Tumor Growth and Metastasis in Papillary Thyroid Carcinoma Cell Lines by Targeting CCND1 and FGF2. *Int. J. Endocrinol.* **2017**, *2017*, doi:10.1155/2017/6180425.

18. Ricarte-Filho, J.C.; Ryder, M.; Chitale, D.A.; Rivera, M.; Heguy, A.; Ladanyi, M.; Janakiraman, M.; Solit, D.; Knauf, J.A.; Tuttle, R.M.; et al. Mutational profile of advanced primary and metastatic radioactive iodine-refractory thyroid cancers reveals distinct pathogenetic roles for BRAF, PIK3CA, and AKT1. *Cancer Res.* **2009**, *69*, 4885–4893, doi:10.1158/0008-5472.CAN-09-0727.

19. Hou, P.; Ji, M.; Xing, M. Association of PTEN gene methylation with genetic alterations in the phosphatidylinositol 3-kinase/AKT signaling pathway in thyroid tumors. *Cancer* **2008**, *113*, 2440–2447, doi:10.1002/cncr.23869.

20. Donehower, L.A.; Soussi, T.; Korkut, A.; Liu, Y.; Schultz, A.; Cardenas, M.; Li, X.; Babur, O.; Hsu, T.K.; Lichtarge, O.; et al. Integrated Analysis of TP53 Gene and Pathway Alterations in The Cancer Genome Atlas. *Cell Rep.* **2019**, *28*, 1370-1384.e5, doi:10.1016/j.celrep.2019.07.001.

21. Ibrahimpasic, T.; Ghossein, R.; Shah, J.P.; Ganly, I. Poorly Differentiated Carcinoma of the Thyroid Gland: Current Status and Future Prospects. *Thyroid* 2019, *29*, 311–321.

22. Zheng, X.; Jia, B.; Tian, X.T.; Song, X.; Wu, M.L.; Kong, Q.Y.; Li, H.; Liu, J. Correlation of reactive oxygen species levels with resveratrol sensitivities of anaplastic thyroid cancer cells. *Oxid. Med. Cell. Longev.* **2018**, *2018*, doi:10.1155/2018/6235417.

23. Ugolini, C.; Basolo, F.; Proietti, A.; Vitti, P.; Elisei, R.; Miccoli, P.; Toniolo, A. Lymphocyte and immature dendritic cell infiltrates in differentiated, poorly differentiated, and undifferentiated thyroid carcinoma. *Thyroid* **2007**, *17*, 389–393, doi:10.1089/thy.2006.0306.

24. Ryder, M.; Ghossein, R.A.; Ricarte-Filho, J.C.M.; Knauf, J.A.; Fagin, J.A. Increased density of tumor-associated macrophages is associated with decreased survival in advanced thyroid cancer. *Endocr. Relat. Cancer* **2008**, *15*, 1069–1074, doi:10.1677/ERC-08-0036.

25. Cherkaoui, G.S.; Guensi, A.; Taleb, S.; Idir, M.A.; Touil, N.; Benmoussa, R.; Baroudi, Z.; Chikhaoui, N. Poorly differentiated thyroid carcinoma: A retrospective clinicopathological study. *Pan Afr. Med. J.* **2015**, *21*, 1937–8688, doi:10.11604/pamj.2015.21.137.6720.

26. Greco, C.; Brigante, G.; Taliani, E.; Corrado, S.; Simoni, M.; Madeo, B. Concomitant medullary thyroid carcinoma with paraganglioma-like pattern and papillary thyroid carcinoma. *Endocrinol. Diabetes Metab. Case Reports* **2019**, *2019*, doi:10.1530/EDM-19-0094.

27. Accardo, G.; Conzo, G.; Esposito, D.; Gambardella, C.; Mazzella, M.; Castaldo, F.; Di Donna, C.; Polistena, A.; Avenia, N.; Colantuoni, V.;

et al. Genetics of medullary thyroid cancer: An overview. *Int. J. Surg.* 2017, *41*, S2–S6.

28. Al-Rawi, M.; Wheeler, M.H. Medullary thyroid carcinoma - Update and present management controversies. *Ann. R. Coll. Surg. Engl.* 2006, *88*, 433–438.

29. Owen, C.; Constantinidou, A.; Miah, A.B.; Thway, K.; Fisher, C.; Benson, C.; Zaidi, S.; Messiou, C.; Van Der Graaf, W.T.A.; Jones, R.L. Synovial sarcoma of the thyroid gland, diagnostic pitfalls and clinical management. *Anticancer Res.* **2018**, *38*, 5275–5282, doi:10.21873/anticanres.12853.

30. Sharma, A.; Kaur, S.; Sarkar, M.; Sarin, B.C.; Changotra, H. The AGE-RAGE Axis and RAGE Genetics in Chronic Obstructive Pulmonary Disease. *Clin. Rev. Allergy Immunol.* 2020.

31. Chmielik, E.; Rusinek, D.; Oczko-Wojciechowska, M.; Jarzab, M.; Krajewska, J.; Czarniecka, A.; Jarzab, B. Heterogeneity of Thyroid Cancer. *Pathobiology* **2018**, *85*, 117–129, doi:10.1159/000486422.

32. Hamamoto, Y.; Kukita, Y.; Kitamura, M.; Kurashige, M.; Masaie, H.; Fuji, S.; Ishikawa, J.; Honma, K.; Wakasa, T.; Hanamoto, H.; et al. Bcl-2-negative IGH-BCL2 translocation-negative follicular lymphoma of the thyroid differs genetically and epigenetically from Bcl-2-positive IGH-BCL2 translocation-positive follicular lymphoma . *Histopathology* **2021**, doi:10.1111/his.14378.

33. Kesireddy, M.; Lasrado, S. *Cancer, Thyroid Lymphoma*; StatPearls Publishing, 2020;

34. Cho, J.K.; Woo, S.H.; Park, J.; Kim, M.J.; Jeong, H.S. Primary squamous cell carcinomas in the thyroid gland: An individual participant data meta-analysis. *Cancer Med.* **2014**, *3*, 1396–1403, doi:10.1002/cam4.287.

35. Tan, L.-C.; Yu, P.-C.; Wang, J.; Zhou, X.-Y.; Ji, Q.-H.; Wang, Y.-L. BCOR-CCNB3 rearranged sarcoma arising in neck misdiagnosed as thyroid cancer: A case report. *Oral Oncol.* **2021**, 105290, doi:10.1016/j.oraloncology.2021.105290.

36. De Felice, F.; Moscatelli, E.; Orelli, S.; Bulzonetti, N.; Musio, D.; Tombolini, V. Primary thyroid angiosarcoma: A systematic review. *Oral Oncol.* 2018, *82*, 48–52.

37. (No Title) Available online: https://ar.iiarjournals.org/content/anticanres/35/10/5185.full.pdf (accessed on Apr 26, 2021).

38. Wygoda, A.; Rutkowski, T.; Szcześniak-Kłusek, B.; Mrochem-Kwarciak, J.; Jędrzejewska, M.; Składowski, K. Primary squamous-cell thyroid carcinoma — A successful treatment with five-year follow-up. *Endokrynol. Pol.* **2017**, *68*, 592–596, doi:10.5603/EP.a2017.0048.

39. Ibrahim, M.I.S.; Jusoh, Y.R.; Adam, N.N.; Mohamad, I. Primary squamous cell carcinoma of the thyroid gland. *Iran. J. Otorhinolaryngol.*

2018, *30*, 65–68, doi:10.22038/ijorl.2017.19732.1663.
